# Microbiota of Cheese Ecosystems: A Perspective on Cheesemaking

**DOI:** 10.3390/foods14050830

**Published:** 2025-02-27

**Authors:** Erasmo Neviani, Monica Gatti, Fausto Gardini, Alessia Levante

**Affiliations:** 1 International Dairy Federation—Italian Committee, 20135 Milano, Italy; erasmo.neviani@unipr.it; 2 Department of Food and Drug, University of Parma, 43124 Parma, Italy; alessia.levante@unipr.it; 3 Department of Agricultural and Food Sciences, University of Bologna, 40127 Bologna, Italy; fausto.gardini@unibo.it

**Keywords:** fermented food, lactic acid bacteria, microbial communities, cheese microbiota

## Abstract

This review contributes to the knowledge on the complex and adaptive microbial ecosystems within cheese, emphasizing their critical role in determining cheese quality, flavor, and safety. This review synthesizes the current knowledge on the microbial interactions and the dynamics of lactic acid bacteria (LAB), encompassing both starter (SLAB) and non-starter (NSLAB) strains, which are pivotal to the curd fermentation and ripening processes. The adaptability of these microbial consortia to environmental and technological stressors is explored, highlighting their contributions to acidification, proteolysis, and the development of distinctive organoleptic characteristics. Historical and technological perspectives on cheesemaking are also discussed, detailing the impact of milk treatment, starter culture selection, and post-renneting procedures on microbial activity and biochemical transformations. This review underscores the importance of microbial diversity and cooperative interactions in fostering ecosystem resilience and metabolic functionality, and it addresses the challenges in mimicking the technological performance of natural starters using selected cultures. By understanding the ecological roles and interactions of cheese microbiota, this review aims to guide improvements in cheese production practices. Additionally, these insights could spark the development of innovative strategies for microbial community management.

## 1. Microbial Interactions in Ecosystems: A Premise

The recent progress in microbiological sciences has exposed the existence of widespread biodiversity in the agri-food ecosystem. Complex microbial communities exist everywhere in nature and, over the years, microorganisms have been mutating and evolving to adapt to ever-changing ecosystems [1,2].

The ability of microorganisms to resist different stress factors induces their resilience in hostile conditions for growth and metabolism. Microbial *consortia* quickly adapt when introduced to a new environment. However, microbial populations might continue to evolve indefinitely, albeit slowly, even in a constant environment, thanks to the contributions of individual mutations to fitness improvement [3,4,5]. Microbial populations gain an advantage from the formation of variant subpopulations—arising from both phenotypic heterogeneity and genotypic variations (e.g., genome rearrangements and mutations)—which may be better adapted to withstand environmental perturbations and exploit novel ecological niches [6]. In this framework, the observation that Charles Darwin shared with Asa Gray can be applied to microbial communities. Darwin said, “What a trifling difference must often determine which shall survive, and which shall perish!” [7]. In procaryotes, intercellular communication and multicellular coordination are widespread and influence the expression and intensity of multiple phenotypes [6,8,9,10,11,12]. In complex ecosystems, these interactions determine the growth, survival, and death of individuals, which, as a whole, constitute the microbial population in equilibrium with the habitat.

A genetic variation can affect both the host genome and the symbiotic microbial consortium, and then is transmitted to the progeny. In this way, evolution proceeds through both cooperation and competition, working in parallel. Following this approach, the subject of evolution is no longer the individual but becomes the community and this represents an evolutionary aspect of absolute importance.

Most fermentation processes depend on mixtures of microbes, which act in concert. In general, it can be stated that complex microbial consortia perform more complex activities (versatility) and tolerate more variation in the environment (robustness) as compared to pure cultures [9,13,14,15]. Even the interaction with the surrounding environment can be crucial for defining cellular cooperation and the evolution of microbial communities [16,17,18,19,20]. Consequently, each cell that develops in the matrix responds to the simultaneous presence and development of the others in the consortium.

The second key feature is the division of labor between the members of the *consortium*, resulting in an overall output that can only be explained by combining tasks performed by individual members or subpopulations [21]. These interactions play a very important role in the evolution of partners and the recent progress of sequencing technology has allowed researchers to highlight the existence of a great diversity and distribution of these associations. These interactions allow multiple microorganisms to survive even in the presence of limited resources, increasing biodiversity in the community. Many microorganisms live in communities and depend on metabolites secreted by fellow community members for survival. Yet, our knowledge of interspecies metabolic dependencies is limited to a few communities with a small number of exchanged metabolites, and even less is known about cellular regulation facilitating metabolic exchange.

Nutrient-rich and dynamic environments support complex microbial communities in terms of species diversity [22], offering new perspectives on food microbial ecology [23]. On the other hand, nutrient-poor or extreme environments like high-salinity ponds contain microbial communities with a lower species complexity [24]. Interestingly, the latter microbial communities still display a high degree of intraspecies diversity (microdiversity). Population heterogeneity is thought to be linked with resilience against environmental uncertainty [25], and mobile genetic elements are the primary sources of heterogeneity [26].

In closely related strains, intraspecies diversity resulting from functionally adaptive traits encodes genomic islands acquired through horizontal gene transfer. Thus, fluctuations in plasmid content among natural communities give rise to subpopulations that offer selective advantages in the face of environmental uncertainty [27]. It is also known that complex bacterial cultures show a higher degree of resistance against bacteriophage attack as compared to simplified selected cultures. This phenomenon can be attributed to the presence of bacteriophages within complex starter cultures, where they play an essential role as part of the microbial community, driving the generation of genetic and phenotypic diversity in the microbial population [27,28]. Analogously, bacteriophages have a regulatory role in population dynamics through density-dependent predation [29]. In this context, the “kill the winner” model is intriguing. It explains how the strong duplication of a specific strain within a microbial community triggers a corresponding increase in viral populations that target that strain. This results in a reduction in the population size of the rapidly growing strain, ultimately promoting diversity within the overall community [27]. However, bacteriophage infection is recognized for causing slow or failed fermentation, which has pushed the need for robust starter cultures. In this perspective, understanding host–phage interactions and their modulation of microbial communities is essential, although complex. A combination of culturomics and metagenomics approaches can integrate our knowledge, and help in developing factory-specific monitoring methods and resilient fermentation cultures [30].

Even the presence of isogenic bacteria can contribute to the survival and evolution of complex microbial ecosystems. In bacterial isogenic variants, although the cells contain the same genetic material, their protein levels can vary due to stochastic events associated with gene expression and regulation [31,32]. Thus, cell-to-cell heterogeneity has important implications, allowing populations of cells to diversify to survive environmental stress, according to the proposed theory of division of labor [33,34].

Finally, we cannot overlook the phenomenon, characterized by non-specific interactions, in which one species inhibits the growth of another. According to this phenomenon called the “Jameson Effect”, the control of the growth inhibition of species in co-culture can be achieved by regulating concentrations and growth rates, allowing a species to reach the stationary phase sooner [35].

Considering this scenario, it becomes clear that the functionality of complex microbial ecosystems depends not only on the abundance and diversity of species, biotypes, and variants, but also on the nature of the interactions among them. These interactions are critical for adaptation to stress, survival, evolution, and the expression of diverse phenotypes [1,10,22,36,37]. Consequently, even multicellularity was proposed as a possibility for understanding the growth and development of complex prokaryotic ecosystems, and the hypothesis of thinking of complex microbial ecosystems as multicellular organisms, whose individual components interact and condition each other, remains stimulating [11,20].

In summary, the interactions among microorganisms, rather than merely their quantity or the presence of various species, biotypes, and variants, often play a crucial role in understanding the development and biological functionality of complex microbial ecosystems.

## 2. Fermented Food Microbiota

Fermentation is one of the oldest technologies used to preserve foods, enhance shelf life, and improve products’ flavor and organoleptic properties. Over time, people have learned to improve, rationalize, standardize, and repeat food fermentation processes [38]. Consequently, fermented foods have become a significant part of the diet in many cultures, and, over time, fermentation has been linked to numerous health benefits [39,40,41,42,43,44,45].

Most food fermentation processes rely on mixtures of microorganisms, including various genera, species, biotypes, and variants, which act in concert and/or at different times to give rise to biochemical modifications, useful for producing the desired product characteristics in terms of texture, flavor, and taste. The interactions that occur within the ecosystem can play a decisive role in the evolution of all the partners present in the fermented food ecosystem itself. From this evidence arises the interest in exploring in depth the diversity of the microbial community involved in natural food fermentation processes and the link with the technological selection induced by food processing.

Microbial selection guides the food fermentation process and represents a crucial step that allows, starting from similar or identical matrices, for the development of even very different products [16,18,19,37,46,47,48,49,50].

The technological processes for producing fermented foods usually imply conditions that guide fermentation through the imposition of differently selective or elective conditions on the microbiota present. The dynamics of the growth, survival, and biochemical activity of the microorganisms in food are the result of stress reactions in response to physical and chemical changes in the food microenvironment due to microbial metabolic activity.

Fermented food production typically imposes distinct selective (or elective) pressures on the resident microbiota, thereby directing the fermentation process. The growth, survival, and metabolic activities of these microorganisms are intertwined with their stress responses to physical and chemical changes in the food microenvironment, many of which are induced by microbial metabolism.

In natural food systems, both stimulatory and inhibitory interactions among microorganisms help preserve a viable core population even amidst continuous changes in the food environment, including those caused by the microorganisms’ own metabolic activity. Overall, microbial consortia in food systems are more versatile and robust than the pure cultures typically used in fermented food production, as they tolerate environmental variations better.

## 3. Cheese Microbiota

The contribution of cheese microbiota to the quality of cheese is of critical significance, as many of the final characteristics of a cheese are due to the complex dynamics and interactions between the cheese’s microorganisms and growth substrates due to the different components of the milk and cheese environment [51].

The earliest historical records of cheese production come from ancient texts discovered in Iraq, dating back to around 3200 BC [52]. It appears that the art of cheesemaking originated in Asia Minor and spread to Europe, where it was further developed by the ancient Romans, who disseminated it throughout the Empire, eventually reaching northern Europe. It is probable that the transformation of milk into cheese occurred independently among different peoples and in various regions of the world even earlier, driven by random experimentation. These practices have been reproduced empirically over time, finally evolving into an industrial process through the acquisition of scientific and technological knowledge, which today is the basis of current cheese production. [38,53,54,55,56,57,58].

Cheese is historically defined as the product that is obtained from the concentration of protein of whole, partially skimmed, or skim milk, or from cream coagulated by lactic acid bacteria and/or rennet. This simple definition is common to a wide variety of cheese typologies [56,58].

Milk is a rich and very attractive substrate for different microbial species able to use lactose as a carbon source. Milk is basically composed of water, fat, casein, whey proteins, lactose, and mineral salts, present in a very different ratio according to the different types of milk. Milk is the result of a balance of three phases: (i) a fat emulsion in water, in which fat globules are dispersed in a “serous” continuous phase; (ii) a colloidal suspension of organized casein in micelles, globular proteins, and lipoproteins; (iii) a lactose solution, soluble whey proteins and enzymes, mineral salts, and vitamins [55,56,58,59,60].

Environmental factors are crucial in shaping the composition of milk microbiota [38,55,60,61,62,63]. Recent advancements in cheese microbiome research have provided a more comprehensive understanding of the bacteria involved in cheese production and ripening [51,64,65,66,67,68]. The cheese microbiome is primarily dominated by lactic acid bacteria (LAB), which belong to the order Lactobacillales and produce lactic acid as the main product of carbohydrate fermentation.

LAB are responsible for cheese curd and cheese fermentation processes and for the modifications associated with cheese ripening. In each cheese, they are present in different numbers, genera, species, and biotypes. LAB from starter cultures (SLAB) added during the production process are typically culturable and abundant in the early stages of cheese production and ripening. In contrast, non-starter LAB (NSLAB), introduced as secondary cultures or naturally present in raw milk, become dominant and essential in the later stages of ripening, but are more challenging to culture [51,69,70,71].

LAB communities in cheese can originate from raw milk, starter cultures, adjunct cultures, and the environment of the cheesemaking plant, including the equipment and feed. This microbial diversity is often enriched by other microorganisms, such as yeasts, molds, and non-LAB. These organisms interact with LAB, contributing to proteolysis and, in some cases, partial lipolysis, which play crucial roles in flavor and texture development in cheese. For cheeses made with pasteurized milk and selected starters, the microbial ecosystem development is simplified, though the biochemical processes remain essentially the same, with their intensity influenced more by the duration and conditions of ripening.

Numerous and diverse factors shape the assembly of the lactic acid bacteria (LAB) community, comprising both SLAB and NSLAB, in cheese [72]. Dairy fermentation, particularly curd fermentation in cheese production, is a dynamic process characterized by fluctuating and often adverse conditions for the fermenting organisms. Throughout cheese manufacturing, environmental parameters such as temperature, pH, osmolarity, and lactose concentration undergo significant changes [41,51,53,64,69,70,73,74,75].

Specific cheese microbes or consortia are adapted to various abiotic stresses, including changes in pH, salinity, temperature, and moisture, as well as biotic stresses like competition and invasion resistance, both at the individual and community levels [46,53,59,61,65,71,73,75,76,77,78].

Various technologies employed by humans to differentiate cheese varieties create specific substrates, such as variations in the fat and moisture content, distribution of curd granule size through cutting and cooking, and the strength of bonds formed during pressing. These factors induce the selection and development of different species and biotypes that contribute to the diverse derivatives of milk. The effective management of milk fermentation processes necessitates integrating microbiological aspects with transformation technology. Besides coagulation, it is cheese curd fermentation that plays a central role in defining the rheological structure of the future cheese. Lactic acid, derived from lactose fermentation, serves as the primary metabolite but it is the rate and intensity of acidification that plays a crucial role in altering the destabilization and structure of casein micelles. Caseins are in a colloidal suspension in raw milk, and the stability of this suspension relies on the presence of saline bridges and the availability of calcium phosphate in a colloidal state. LAB acidification of the curd can occur at different rates, according to the selected culture, and can lead to the complete or incomplete consumption of lactose. Based on the growth characteristics of the LAB strains, the release of charged molecules resulting from lactic acid fermentation will be different. Therefore, the various modes of acidification (fast or slow, complete or incomplete) resulting from LAB fermentation can alter this physical–chemical equilibrium differently, increasing the permeability of the curd. Varying degrees of acidification promote different curd structures [55,56,57,58,59,79,80].

The structure of the coagulum in the vat, before the breaking process, is influenced not only by the quantity and type of rennet but also by the intensity of acidification in the curdling milk. Acidification and lactose depletion are the initial stages of curd formation when lactic fermentation-induced acidification starts to make the substrate less hospitable and appetizing to most other microbial species. Therefore, rapid acidification and growth from SLAB are paramount to minimizing the risks of spoilage and process failure.

During the initial stage of cheesemaking, the mild proteolytic activity of NSLAB promotes an increase in the amount of free amino acids in milk. These free amino acids or small peptides are crucial for facilitating the rapid growth of SLAB and accelerating the kinetics of acidification [81,82]. Consequently, these metabolic interactions between NSLAB and LAB are crucial for inhibiting spoilage bacteria and contributing to curd structure formation [55,56,57,58,59,79,80]. Recent studies in different microbial species have shown that high growth rates come at the expense of the expression level of metabolic enzymes and/or stress proteins [69,83,84]. In starter cultures, such a trade-off would affect flavor formation, which depends on the level of flavor-forming enzymes and the prolonged survival of cells.

Also, the structure of the curd after it is removed from the vat is primarily influenced by acidification resulting from LAB activity. LAB’s acidification activity within the curd depends on factors such as the remaining sugar availability, pH, residual moisture, and curd temperature. These parameters can vary across different areas of the curd or fresh cheese. In larger cheeses, significant differences in acidification activity are typically observed between the central and outer parts of the cheese. As a result, large cheeses may exhibit varying rates of ripening in different zones [81,85,86].

The spatial distribution of microbes within a curd block is driven by the cheese’s intrinsic (e.g., the availability of substrates and co-factors, presence of inhibitor/activator compounds, pH, a_w_, and redox potential) and extrinsic (e.g., oxygen availability, temperature, and relative humidity) factors [87]. After milk stirring and coagulation, a relatively uniform but stochastic distribution determines the bacterial immobilization in the curd. Such immobilization affects the spatial repartition of colonies, and creates microscopic environmental niches, also subjected to fluctuations throughout space and time [16] (Jeanson et al., 2015).

On average, the number of lactic acid bacteria (LAB) in the curd of various dairy products can reach nearly a billion colony-forming units per gram (cfu/g). Studies on long-ripened cheeses have shown that the counts of starter LAB (SLAB) can remain high for several months during cheese ripening, although different species and biotypes may succeed over time [73,88,89]. This large population of bacteria represents a significant reservoir of enzymatic activity because their breakdown and the release of intracellular enzymes are crucial factors in cheese ripening [18,69,73,88,90,91,92,93,94,95,96,97,98].

In long-ripened cheeses, it has also been observed that the autolysis of SLAB can provide sufficient nutrients to support the growth of non-starter LAB (NSLAB) in vitro [18,53,73,77,93,99,100,101,102]. To elucidate the metabolic interactions among microorganisms, integrated systems biology is essential for combining perspectives from post-genomics technologies. Future research should focus on variations in cell physiology within microbial populations, as spatial distribution within the cheese matrix creates microenvironments affecting interactions between starter cultures and non-starter lactic acid bacteria (NSLAB) [72]. Modeling microbial communities can enhance the efficiency and reduce the cost of processes like cheese ripening [103].

Time, temperature, and relative humidity conditions, as well as the salt diffusion process within the cheese, evolve during ripening and differently modify microbiota evolution and, consequently, microbial activity and the associated enzymatic activities. This activity, in turn, depends on the type of microbiota present in the curd and the type and dose of rennet used. In this frame, proteolysis against caseins should indeed be considered a crucially determining event. As a result of the enzymatic activities (primary metabolism and proteolysis), microorganisms favor the biochemical processes which transform curd to mature cheese.

## 4. The Cheesemaking Process and Associated Microbial Stresses

During the transformation of curdled milk and the initial hours of maturation, lactic fermentation and microbial proliferation play crucial roles. However, in the later stages of ripening, as lactose depletion occurs, microbial death and lysis release a significant amount of intracellular proteolytic enzymes. These enzymes, some of which remain active in the curd for extended periods, contribute to various stages of cheese ripening [87].

The transition from curd to cheese begins with a reduction in the moisture content due to whey draining. This transformation is primarily an enzymatic process, where major components of the curd are broken down into simpler substances. Throughout this process, viable microorganisms undergo growth and eventual death, exerting a pivotal influence on both structural and sensory changes in the curd and cheese paste.

To comprehend how production technology shapes the growth and metabolic activity of microorganisms in cheese, it is beneficial to segment the cheesemaking process into three distinct events, each characterized by a modified or evolved microbiota (Table 1):

(a) The arrival of the milk at the dairy.
(b) The processing of the milk in the vat.
(c) Post-renneting operations outside the vat.

Upon arrival at the dairy plant, raw milk harbors a diverse microflora influenced significantly by milking procedures, timing, and collection methods (Bettera et al., 2023) [63].

(a) The arrival of the milk at the dairy.

Due to its nutrient richness, milk’s near-neutral pH and favorable temperature create an ideal substrate for various microbial species, allowing only those capable of thriving in this environment to colonize it. Among these, there is the *Lacticaseibacillus* genus, which plays a crucial role in dairy production as NSLAB [105]. LAB and the entire raw milk microbiota are subjected to various stresses due to high- or low-temperature treatments. Firstly, to maintain the microbiological quality of milk and delay the growth of harmful or spoilage bacteria, milk can be cooled to different refrigerated temperatures at the farm [75,106,107]. Refrigeration encourages the development of psychrotrophic bacteria while partially inhibiting the growth of mesophilic and thermophilic LAB. Additionally, milk can be thermized (heated for at least 15 s at temperatures between 57 °C and 68 °C) or pasteurized (heated to at least 72 °C for 15 s or any other equivalent combination) to prevent foodborne diseases and spoilage. These treatments reduce certain microbial species depending on the time/temperature combination used and significantly decrease psychrotrophic and mesophilic populations, including those of non-starter LAB (NSLAB) [56,58,59,80].

Other factors that can reduce the number and biodiversity of LAB in milk upon arrival at the dairy include skimming treatments. Skimming milk through natural cream rising [53] reduces the number of spore-forming bacteria, which are carried into the cream by fat globules. Depending on the temperature and duration of natural cream separation, there may be an increase in the mesophilic LAB present in raw milk [73].

Centrifugal skimming also can impact the milk microbiota by selecting different LAB species, favoring some over others, and resulting in a decrease in viable lactobacilli in ripened cheeses [108]. Consequent to its prior technological ‘history’, the residual microbiota of raw milk reaching the vat may be dramatically different from that present immediately after milking, both in terms of the bacterial count and species and biotype composition.

On the contrary, a technological option to increase the number of LAB in the milk before cheese processing is pre-maturation. This process prepares milk for cheese manufacturing by adding commercially selected LAB as an adjunctive culture. Pre-maturation occurs at a temperature below the optimal growth range for the LAB strains used. The primary effect of pre-maturation is partial proteolysis, which produces low-molecular-weight peptides that benefit the starter bacteria added later, thereby accelerating the subsequent acidification process [53,59,73,80].

(b) The processing of the milk in the vat

In this environment, several technological phases affect the development of the cheese microbiota. One such step, the addition of a starter, causes an increase in lactic acid bacteria. Other steps, such as the possible addition of additives or the possible cooking of the curd, cause a selection of the LAB that will be found in the curd extracted at the end of the first phase of the milk-to-cheese transformation. But even less direct phases, such as the addition of rennet and the breaking of the curd, can influence the distribution of lactic acid bacteria because they affect the different distribution of nutrients between the liquid phase of the whey and the solid phase of the curd [56,58,59,80].

The addition of starter cultures initiates lactic fermentation, leading to milk and curd acidification, and increases the LAB population, directing fermentation based on the technology used. This practice, aimed at reducing spoilage and pathogenic microorganisms in cheese, is now common in industrial production. Starter LAB (SLAB) are divided into homofermentative mesophilic and thermophilic cultures. Thermophilic SLAB are typically used in the production of semi-hard, hard, and extra-hard cheeses (e.g., Italian and Swiss varieties), where curd cooking is part of the process. Starters can also be arbitrarily divided into natural LAB cultures of indefinite composition produced daily by the dairy, and selected LAB cultures of defined composition usually produced by a specialized industry [53,58,59,80].

The use of natural starter cultures is characteristic of traditional artisanal cheese production. There are two main types: whey starters and milk starters. Natural whey starters are made by incubating the residual whey from cheesemaking at a selected temperature. When curd cooking is involved, the heat treatment selects the microbiota of the whey starter, shaping the microbiological ecosystem of the vat milk. The whey left after manufacturing has a unique temperature, chemical composition, and microflora, varying by species and biotypes depending on the chosen technology and type of milk [21,59,73,76,80,109,110,111,112]. Natural milk starters are obtained by thermal selection from the raw milk microflora. Briefly, the starter is produced by heating raw milk at 60–65 °C for 15 to 30 min, followed by rapid cooling to the incubation temperature; the incubation conditions complete the selection and are different depending on the type of cheese to be produced. Starter culture communities may face several selective pressures during starter fermentation. Mixed-strain undefined cultures result in more resilience and robustness in dairy processes as compared with selected ones, characterized by a low strain diversity [53,113].

The biological complexity of the LAB in natural starters is both their strength and weakness [8]. The complexity and biodiversity ensure adaptability to artisanal production technologies, but the variety of species and biotypes makes daily propagation more challenging to standardize.

Selected starters are LAB isolated from natural habitats and chosen based on technological characterization tests, which can be conducted on individual strains or strain mixtures. Their use in the dairy industry often involves the heat treatment of raw milk and the need for standardized processing times. Unlike natural starters, selected starters ensure the reproducibility of the technological performance required by industrial production. To reduce the incidence of phage infections, selected starters undergo rotations, alternating strains with similar technological capabilities but different sensitivities to bacteriophage infection. Currently, selected starters are well-studied LAB in terms of strain characterization and technological performance.

To address the technological issues caused by unwanted spoilage microorganisms, aids or additives may be added to the milk in certain cheesemaking processes. These aids or additives limit, delay, or stop the growth of undesired bacteria but can sometimes also alter the growth activity of LAB [56,74,114].

Coagulation represents the first structural transformation of milk from liquid to gel. For most cheeses, coagulation is primarily enzymatic, causing the destabilization of casein due to demineralization. Casein destabilization can also be caused by the fermentative activity of LAB. In different cheesemaking processes, these two phenomena contribute to varying extents. Generally, enzymatic coagulation produces less demineralized and more elastic curds and cheeses (e.g., Emmental), while predominantly acidic coagulation, characterized by greater lactic bacteria activity, results in more demineralized and friable curds.

Additionally, rennet enzymes can contribute to the proteolysis of curd/cheese proteins during ripening, reducing the peptide size and making them more accessible to the proteolytic activity of LAB during aging. The thermal treatment of the curd can inhibit this secondary activity [55].

The phase following the coagulation of casein, which forms the curd, is its breaking. This involves dividing the curd into pieces of the desired size (from a few centimeters to a few millimeters) to increase the surface area for whey separation. Breaking the curd is crucial as it determines the quality (fat content and fine curd particles) and quantity of separated whey, as well as the kinetics of its separation. The amount of whey remaining inside the curd is significant from a microbiological perspective because it defines the quantity of soluble fractions, particularly lactose and small amounts of peptides, that will remain available for bacterial development.

Curd cooking is a heat treatment used in the production of some cheeses called “cooked” cheeses. This process directly affects separation and drainage by significantly increasing the curd’s syneresis capacity. The intensity of the heat treatment is generally related to the desired residual moisture content in the cheese and is also associated with the degree of curd rupture. From a microbiological perspective, cooking, which selects the more thermophilic LAB, influences the early stages of the fermentation and ripening of the curd and cheese. The heat treatment induces the thermal selection of LAB also naturally present in the milk. At this stage, there is a close relationship between the technology used and the selection of the dominant microbiota. The curd heat treatment step primarily shapes the bacterial community in the curd, at least during the initial stages of ripening.

The microbiota in the liquid form of milk in the vat is evenly distributed, but in the semisolid form of curd, it is divided between the cells adhering to the curd and those dispersed in the liquid whey phase.

(c) Post-renneting operations outside the vat

When the curd is extracted from the vat, this division becomes even more pronounced, and it will be the cells that have the best ability to adhere to the curd structure that will have the greatest chance of contributing to defining the characteristics of the final cheese. The size and shape of the cheese are dramatically affected by all of the factors defined throughout the production phases ‘outside the vat,’ which regulate the replicative capacity of LAB and the entire cheese microbiota.

Once extracted, the curd undergoes several operations that determine its transformation into different cheeses. From a curd without a defined structure that has a predominant milk flavor, cheeses characterized by various shapes, consistencies, tastes, and aromas are obtained. This transformation takes place through four main phases (molding, acidification, salting, and ripening) that can vary in time and manner, which, even if minimal, can differently regulate the contribution of the cheese microbiota as a microbial community that, in its complexity, follows the growth curve from the lag phase to the death phase, up to cell lysis. In these phases, LAB (starter and non-starter) may interact with other non-lactic bacteria, yeasts, and filamentous fungi, depending on the type of cheese.

The curd, just after separation from the whey, is generally still unable to maintain its own shape, even though it may have some degree of cohesion. The mold provides temporary support (from a few hours to 24–48 h), allowing for the consolidation of the cheese structure due to the combined actions of whey drainage and progressive cooling of the curd. Molding directly influences microbial metabolisms, as the shape and size determine the spontaneous cooling rate of the curd, thus affecting microbial growth rates and the desired acidification of the curd due to the growth of SLAB. Acidification, in turn, decreases the hydration of casein micelles and progressively demineralizes them, increasing the permeability of the curd and facilitating the completion of whey separation. The transformation of the curd into cheese is of paramount importance from the microbiota perspective. During this technological phase, which can last from a few hours to one day and precedes salting, curd acidification and whey drainage are largely completed. Depending on the size of the cheese, a thermal gradient forms in the curd, which may favor the different development of species and biotypes of the LAB population.

Curd salting marks the transition between curd and cheese in the dairy process. Salting imparts savory notes to the cheese, facilitates the completion of whey drainage, and promotes rind formation. Finally, by reducing water activity, the presence of salt causes microbial selection, depending on the amount of salt absorbed by the cheese.

Although curds for different cheese varieties are recognizably different at the end of manufacture, the peculiar characteristics of cheeses develop during ripening. In most cases, the biochemical changes that occur during ripening, and hence the flavor, aroma, and texture of the mature cheese, are largely predetermined by the manufacturing process. During ripening, an extremely complex set of biochemical changes occur through the catalytic action of the following agents: coagulants; indigenous milk enzymes, especially plasmin and lipoprotein lipase, which are particularly important in cheese made from raw milk; starter bacteria (SLAB) and their enzymes; and secondary microbiota (NSLAB) and their enzymes [115,116,117]. Additionally, different temperatures in various areas of the cheese (from the rind to the core), which are maintained for different times depending on the cheese’s size, can regulate the degree of autolysis of the microorganisms present. Consequently, different areas of the cheese will experience fermentative events or proteolysis of varying intensity based on how the lactic microflora develops during acidification.

## 5. Conclusions

The ability of microorganisms, like all living species, to colonize an ecosystem depends on their capacity to interact with other species within the same ecosystem and to adapt and integrate into the evolving environment. The fermentation of foods is a prime example of this phenomenon and is crucial for understanding the composition and technological functionality of their characteristic microbiota.

Thus, the colonization of fermented food by different microorganisms can be examined in terms of both ecological strategy and community development, and can be discussed within the context of complex microbial communities [1,10,21,37,47,50,53,118,119,120,121].

The interactions within the food ecosystem can significantly influence the evolution of all organisms present in the ecosystem. This has led to increased interest in studying the diversity of fermenting microbial communities and linking their evolution to their ability to adapt to technological processes and product quality.

In sourdough microbiota, it has been observed that microbial communities undergo compositional changes that challenge their resilience. For resilience and optimal performance, the sourdough metacommunity includes dominant, subdominant, and satellite players, which together ensure gene and transcript redundancy [122].

Also, in natural whey starter (NWS) used to produce Italian long ripened cooked cheeses, it has been observed that an LAB consortium coevolved during adaptation to whey and curd acidification [109]. In their study of NWS used to produce Parmigiano Reggiano cheese, Bertani and colleagues observed the significant presence of microorganisms in the natural whey starter culture mixed with raw milk. These microorganisms primarily originate from raw milk, forming a core microbiota essential for the ecosystem’s persistence. This core adapts to the mixture of raw milk and whey, whether non-acidified or acidified. A minority of these microorganisms can also adapt to the curd ecosystem, a crucial aspect for the cheesemaking process. Although the natural whey starter provides optimal growth conditions for many bacteria (species and/or biotypes), only a small portion can effectively adapt to the curd ecosystem compared to the cooked non-acidified whey and natural whey starter ecosystem. It can be assumed that a core microbiota supports the ecosystem’s persistence, adapting to the mixture of raw milk and natural whey starter in the vat, to sweet whey after curd separation, or to acidified whey after natural whey starter preparation. The majority of LAB present in acidified whey are well adapted to this substrate, ensuring that they effectively reach the desired level of acidification.

It is possible to speculate that the management of this dynamic ecosystem must consider likely a “super-organism”, consisting of the total microbial metabolism and interactions between individual microbes [8]. This kind of super-organism activity reflects the multicellularity concept proposed by Shapiro [11,20], which is crucial for understanding the growth and development of complex prokaryotic ecosystems, where individual components interact and influence each other. Thanks to new methods, such as shotgun metagenomics, we can reveal the functional properties of these cultures. In the case of Swiss hard cheese starter cultures, metagenome-assembled genomes help with understanding the mutualistic interactions established by strains in food ecosystems, mostly linked to nutrient exchange and phage resistance specificity [28]. The high functional redundancy of strains in these cultures ensures product stability, while strain-level variability provides adaptability to environmental changes of various natures.

A complex and only partially understood network of interactions between biotic and abiotic factors leads to continuous changes in the microbial balance during fermented food production. Simplifying the cheese microbiota, we can categorize the various microorganisms into three groups. The first group includes microorganisms that are only occasionally present and hold little to no technological significance, often acting as occasional contaminants. This group is larger when cheese is produced from raw milk and includes microorganisms likely originating from environmental contamination and not involved in the persistence or resilience of the ecosystem. The second group comprises microorganisms (mainly LAB biotypes and variants) that are essential for the cheese transformation process, driving the biochemical events necessary to convert raw milk into cheese. This core microbial group adapts and evolves throughout the different stages of the cheesemaking process.

The third group consists of microorganisms that, while not essential for cheese manufacture, contribute to specific biochemical events that result in unique organoleptic characteristics. This group is more diverse and abundant in artisanal cheeses and, although not a biological element of stability and continuity in the ecosystem like natural whey starter, is functional in the curd fermentation process and capable of adapting to technological stress factors [8,123,124].

Understanding the interactions between these different parts of the cheese microbiota, rather than merely their presence and abundance, is key to comprehending technological functionality, adaptability to stress, survival, evolution, and phenotype expression in the final cheese product. The challenges in selecting starter cultures with technological and aromatic performances comparable to those achieved with natural starters of undefined composition are likely linked to this complexity. Additionally, minority populations or seemingly non-viable strains may be necessary to maintain essential cellular interactions. This evidence underscores the importance of exploring the diversity of microbial communities involved in dairy fermentation processes and their link to technological aptitude and cheese quality. In the future, the use of increasingly advanced analytical techniques should enable us to understand the various functional and ecological roles of different microbial components within a complex cheese ecosystem. This approach will allow us to differentiate between microorganisms essential for cheese production, those that support the survival and development of the microbial consortium, and those that are merely incidental contaminants.

While culture-independent approaches have expanded our knowledge of complex ecosystems, there is a need to develop new culturomics methods to disentangle these intricate microbiological interactions. A key challenge for future research will be identifying the factors influencing the growth of ‘supporter’ strains, which can indirectly enhance the cheesemaking performance of ‘leader’ strains. Understanding these factors could reveal the mechanisms driving cooperation between these strains. Ultimately, this shift in perspective could help design defined microbial consortia that match, or even surpass, the performance of undefined starters. With this knowledge, we may be able to replicate the technological performance of undefined starters, characteristic of complex microbial consortia, by employing selected cultures.

## Figures and Tables

**Table 1:** Technological steps during cheesemaking and their effect on microbial growth and biochemical events in the cheese. Adapted from [53,59,104].

Cheesemaking Phase	Technological Steps and Effect on Raw Milk Microbiota	Microbial Growth and Biochemical Events
**a. Arrival of the milk at the dairy**		
Raw milk collection	**Microbial contamination** Microorganisms come from the production environment and depend on the milking and milk collection methods Various contaminating microbial populations (notably, mesophilic microorganisms capable of using lactose to grow and trivial contaminants)	Mesophilic LAB growth and partial milk acidification. Other mesophilic bacteria growth
Any heat or non-heat treatments of raw milk before vat operations	**Refrigerated storage** Selective development of psychrotrophic bacteria and decrease in the mesophilic milk microbiota growth rate	Development of psychrotrophic species present as contaminants in raw milk Possible production and release of heat-resistant bacterial proteases and lipases
	**Pasteurization** Elimination of non-spore-forming pathogenic bacteria (at least 5 logarithmic reductions) and reduction in the overall number of bacteria present, including LAB (3-4 orders of logarithmic magnitude)	Numerical reduction in present microorganisms, depending on the thermosensitivity of the different species and biotypes
	**Thermization** Reduction in the total number of viable bacteria initially present in raw milk (effect depending on the time/temperature ratio used), while maintaining viable the majority of the initial raw milk LAB microbiota	Numerical reduction in the proportion of the microorganisms present in raw milk, depending on relation to the thermosensitivity of the different species and biotypes
	**Skimming by centrifugatio** **n**	Modification of milk fat content
	**Skimming by creaming (natural separation of milk fat caused by its lower density; used for Italian Grana Padano and Parmigiano Reggiano)** Raw milk microbiota modification	Reduction in the spores of spore-forming bacteria, particularly *Clostridium* spp. (dragged into the cream by the interaction with surface of fat globules), possible partial increase in the mesophilic microflora present (including LAB) depending on the modalities of the creaming conditions (duration and temperature) Partial milk acidification and modification of casein favoring the subsequent activity of the coagulant
	**Bactofugation** Centrifugation at a high rpm/minute to remove bacteria and spores Separation of the cream, which can be added again at a known quantity to the milk after heat treatment Different temperatures could be used	Separation of spores of spore-forming bacteria and of other different microorganisms depending on physiological structure Reduction in the microorganisms present in raw milk depending on operating time/temperature ratio used
Vat milk operations	**Starter addition:** Natural starter (prepared in milk or in whey) Selected starter	Numerical increase in milk LAB microbiota (thermophilic or mesophilic LAB) pH decrease of milk (only in case of liquid starter utilization) and modification of the casein structure that favors the subsequent activity of the coagulant Start of fermentation and starter LAB species growth and metabolism Lactose reduction Milk pH decrease Inhibition of pathogenic and/or spoilage microorganisms
	**Possible addition of technological aids and/or additives**	Possible different inhibitory activity against some species depending on technological aids or additives used
**b. Processing the milk in the vat**		
Vat milk coagulation	**Rennet addition** Addition when a specific vat milk acidity (milk pH) is reached Possible use of different amounts of rennet Possible use of rennet preparations with different enzymatic compositions (chymosin/pepsin ratio) Use of different time/temperature ratio for renneting	Modification of vat milk structure (liquid to solid) Destabilization of casein micelles Coagulation of milk In relation to the quantity of rennet and the previous acidification of the milk, definition of coagulum/curd structure Possible subsequent participation of rennet enzymes in proteolysis of curd/cheese proteins during ripening as a secondary enzymatic activity Different amounts of small free peptides that can contribute to microbial growth (LAB in particular) Lipolytic activity associated with some rennet preparations (rennet paste) in cheeses that do not undergo curd cooking
	**Vat milk coagulation** Use of different time/temperature ratio for coagulation	Definition of the dominant curd microbiota species (starter and raw milk LAB) Definition of the coagulum rheology, of the coagulum syneresis, and of the chemical and physical properties of the curd
Curd breaking	**Definition of the size and rheology of the curd grains**	Addressing the humidity of the curd and initial definition of the nutrient availability to the curd for bacteria (nutrients in excess at this stage)
Resting the curd under whey and curd cooking under stirring	**Use of different time/temperature ratio for resting under whey before curd cooking** **Use of different time/temperature ratio for curd grains cooking under stirring**	Whey separation from curd Definition of curd grain structure (texture, humidity, pH…) Thermic selection of LAB species, LAB growth, and curd acidification Inhibition of other microorganisms in relation to time/temperature ratio used Inhibition (partial or complete) of secondary rennet enzymatic activities
Curd extraction, separation from whey, and placing into a mold	**Use of different molds to achieve different cheese shapes** **Possible use of different environment temperatures** **Start of curd/cheese cooling**	Definition of cheese shape and dimensions Completion of the whey purging phases and definition of the humidity of the curd Definition of temperature gradients between the surface and interior of the curd Colonization of the different areas of the cheese by the microbiota Development of the starter LAB Selection of the LAB biotypes most tolerant to curd condition Progressive reduction in carbohydrates and decrease in pH Inhibition/competition of pathogenic and/or some anti-dairy microflora Development of secondary microorganisms Initiation of the processes of bacterial autolysis and release of cytoplasmic enzymes
**c. Post-renneting operations outside the vat**		
Salting	**Definition of salt penetration mode as a function of NaCl concentration of brine solution (or NaCl used for dry salting), salting time, cheese size, cheese pH, humidity of the cheese**	Definition of cheese moisture Penetration of salt into the cheese and definition of NaCl content in the different area of the cheese during salting, and as a consequence during cheese ripening Different microbial selection in the different cheese areas characterized by different NaCl concentrations Growth of secondary microbiota (NSLAB and not LAB) Increase in bacterial autolysis and bacterial cytoplasmatic enzyme release in cheese
Ripening	**Definition of final cheese characteristics** **Use of different environmenta** **l (humidity and temperature) conditions for different defined times**	Gradual decrease in cheese humidity Degradation of residual sugars Evolution of cheese microbiota; initial multiplication followed by a gradual deep decrease in cheese microbiota Initial growth of SLAB followed by a gradual deep reduction during ripening Initial growth of NSLAB from raw milk if present in relation to possible raw milk heat treatments before cheesemaking. Decreases during the last part of ripening Growth of secondary microorganisms Increase in bacterial autolysis and bacterial cytoplasmatic enzyme release in cheese Increase in pH Protein and lipid degradation Proteolysis by the following routes: - Plasmin proteolytic activity (negligible or strongly limited for pasteurized milk) - Rennet secondary proteolytic activity (not present if curd is cooked) - Bacterial proteases Degradation of organic acids Possible lipolysis by the following routes: - Lipolytic microorganisms - Rennet in paste form - Lipolytic cytoplasmic enzymes released by bacterial autolysis Production of aroma compounds

## Abbreviations

The following abbreviations are used in this manuscript:

LAB	Lactic acid bacteria
NSLAB	Non-starter lactic acid bacteria
SLAB	Starter lactic acid bacteria
NWS	Natural whey starter
